# The roles and heterogeneity of CD8^+^ T cells in inflammatory bowel disease: A narrative review of insights from single-cell transcriptomics (Review)

**DOI:** 10.3892/ijmm.2026.5801

**Published:** 2026-03-17

**Authors:** Rui Zhong, Jing Guo, Wujie Ye, Zhihui Deng, Huangan Wu, Qin Qi, Guona Li, Lu Zhu, Yan Huang, Luyi Wu

**Affiliations:** 1 Key Laboratory of Acupuncture and Immunological Effects, Yueyang Hospital of Integrated Chinese and Western Medicine, Shanghai University of Traditional Chinese Medicine, Shanghai 200437, P.R. China; 2 Department of Traditional Chinese Medicine Health Preservation, School of Acupuncture-Moxibustion and Tuina, Nanjing University of Chinese Medicine, Nanjing, Jiangsu 210023, P.R. China; 3 Department of Acupuncture and Rehabilitation, Jiangsu Province Hospital of Chinese Medicine, Affiliated Hospital of Nanjing University of Chinese Medicine, Nanjing, Jiangsu 210029, P.R. China

**Keywords:** CD8^+^ T cells, inflammatory bowel disease, single-cell RNA sequencing, biological techniques, immune therapy

## Abstract

The present review investigates the role and characteristics of CD8^+^ T cells in inflammatory bowel disease (IBD) using single-cell transcriptomics, revealing their pivotal functions and remarkable heterogeneity. In IBD, CD8^+^ T cells exhibit marked phenotypic and functional diversity, with distinct subpopulations exhibiting unique signaling pathway activation profiles that associate with varying clinical outcomes. Furthermore, CD8^+^ T cell subsets in IBD participate in complex crosstalk networks involving immune and non-immune cells, modulating inflammatory responses and tissue homeostasis. The present review synthesizes the dynamic complexity of CD8^+^ T cell behavior in IBD and identifies promising therapeutic opportunities through targeted modulation of specific T cell subsets and their interactions within the colonic microenvironment.

## 1. Introduction

Inflammatory bowel disease (IBD) comprises a set of chronic, immune-driven disorders of the gastrointestinal tract, defined by recurrent mucosal inflammation (1,2). IBD, which comprises two major subtypes, Crohn's disease (CD) and ulcerative colitis (UC), has become a global healthcare challenge with steadily rising incidence rates (1), in Southeast Asia alone, the number of cases increased from ~103,884 in 2017 to ~118,000 in 2020, and is projected to reach ~199,000 by 2035 (3).

IBD arises from complex interactions among environmental factors, gut microbiota and immune-mediated mechanisms in genetically susceptible hosts (4). For decades, research on IBD pathogenesis has focused on mucosal immunity, particularly T cell responses. A previous study suggest that dysregulated innate and adaptive immune mechanisms are central to the pathogenesis of uncontrolled intestinal inflammation in IBD (5). Key components of intestinal immunity include intestinal epithelial cells (IECs), macrophages, dendritic cells (DCs), neutrophils, natural killer T (NKT) cells, innate lymphoid cells (ILCs), T cells and B cells (6). As key mediators of adaptive immunity, T cells, including CD4^+^ and CD8^+^ subsets, mature in the thymus, migrate to peripheral tissues and orchestrate humoral and cellular immune responses (7).

**Figure 1:**
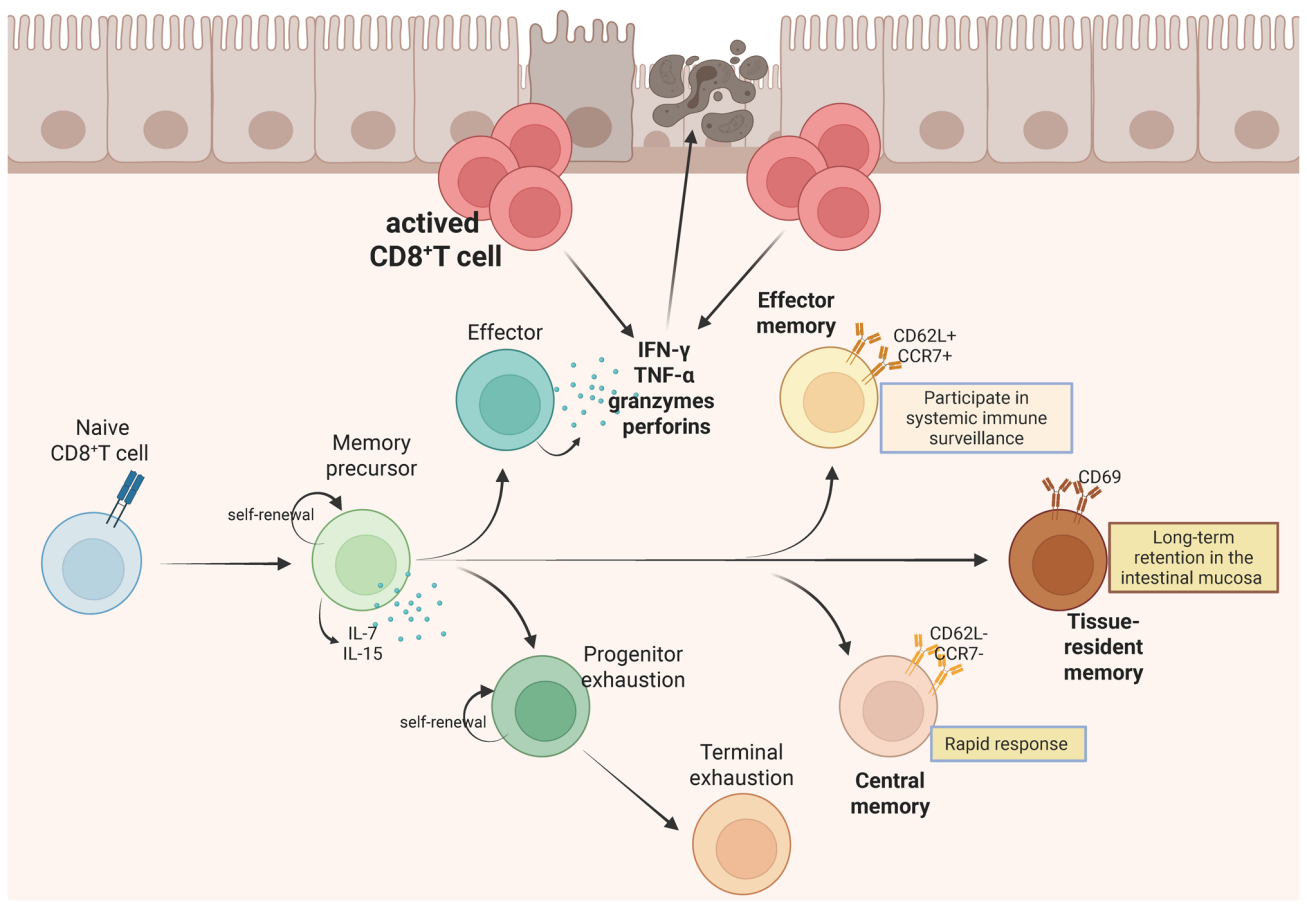
The differentiation and function of CD8^+^ T cells in the colon. The differentiation of naive CD8^+^ T cells into memory subsets progresses through distinct immunological phases, activation, expansion and contraction, under the coordinated regulation of cell surface receptors, soluble factors, transcriptional programs and metabolic adaptations that collectively ensure long-term cellular survival and effector functionality. Importantly, studies on intestinal mucosa of patients with inflammatory bowel disease have consistently demonstrated the presence of activated CD8^+^ T cell populations, implicating their potential role in disease pathogenesis. The figure was prepared using Biorender.com (agreement no. KK29A0WIOJ).

The differentiation of CD8^+^ T cells is a precisely controlled process influenced by antigen exposure characteristics and dynamic changes in environmental factors, particularly the intensity and duration of inflammatory signals. Following antigen encounter within an acute inflammatory context, antigen-specific naive CD8^+^ T cells initiate clonal expansion and commit to a cytotoxic effector lineage. These effector populations secrete proinflammatory cytokines such as IFN-γ and TNF-α and cytotoxic molecules including granzymes and perforin, with most undergoing apoptosis during the contraction phase while a subset persists as memory T cells (8). CD8^+^ T cells are important for immune homeostasis, with studies demonstrating that CD4^+^ T and CD8^+^ T cell subsets are both activated in the peripheral blood and intestinal mucosa of patients with IBD, irrespective of age (9,10). Notably, the colonic lamina propria harbors abundant CD8^+^ tissue-resident memory T (TRM) cells that may drive IBD-associated tissue pathology (11,12). These observations emphasize the important need to elucidate the phenotypic and functional heterogeneity of CD8^+^ T cells in IBD pathogenesis, as such insights could uncover potential targets for novel therapeutic strategies (Fig. 1).

Single-cell RNA sequencing (scRNA-seq) offers distinct advantages over conventional bulk sequencing by enabling high-dimensional analysis of individual cells with complete transcriptome resolution. This technology characterizes cellular composition and population dynamics and detects actively transcribed genes at single-cell or cluster levels, reconstructs cellular differentiation trajectories and subtype evolution, identifies key regulatory genes governing these processes and deciphers intercellular communication networks (13,14). ScRNA-seq, particularly, is valuable for investigating CD8^+^ T cells, a functionally heterogeneous population and the comprehensive nature of the technology facilitates cross-tissue/cross-species cell type identification and, through multimodal analytical approaches, reveals previously unrecognized receptor-ligand interactions that may inform therapeutic target discovery (15,16). The role of CD8^+^ T cells in IBD has remained controversial for decades. While some studies have identified their anti-colitic properties through regulatory mechanisms, others have implicated specific subsets in driving tissue inflammation and epithelial damage (10,17,18). These conflicting findings may stem from their tissue-specific origins (for example, peripheral blood mononuclear cells, intestinal epithelium or lamina propria) and intrinsic heterogeneity among CD8^+^ T cell subsets, which exhibit profound phenotypic and functional diversity (19-21). Present investigations are predominantly centered on conventional cytotoxic CD8^+^ T cells and CD8^+^ regulatory T cells (CD8^+^ Tregs) (22,23), with additional attention given to non-classical subsets such as type II cytokine-producing CD8^+^ T cells, as well as CD8^+^ populations secreting IL-9, IL-17 and IL-22 (21). The conventional approach of RNA-seq, which is limited to analyzing specific cell types or tissues under the assumption of population homogeneity, may represent one key factor contributing to the current controversies in CD8^+^ T cells research (20). By contrast, scRNA-seq provides comprehensive transcriptome profiling at single-cell resolution, enabling high-resolution examination of cellular heterogeneity within precisely defined and well-characterized cell populations (24,25). ScRNA-seq of the colonic tissues of patients with IBD has identified highly heterogeneous CD8^+^ T cell populations exhibiting distinct phenotypic and functional characteristics (19,20).

## 2. Search strategy and selection criteria

The literature search for the present review was conducted in the PubMed (https://pubmed.ncbi.nlm.nih.gov/), Web of Science (https://www.webofscience.com), Embase (https://www.embase.com), and the Cochrane Library (https://www.cochranelibrary.com/) databases for articles published from January 1, 2015 to December 31, 2025, with no language restrictions. A detailed description of the search strategy is provided in Table SI. To ensure a comprehensive capture of the relevant literature, the reference list of all included articles was manually screened for additional pertinent studies. Two reviewers (QQ and GL) independently screened titles, abstracts and full texts using the following inclusion criteria: i) Articles with human patients with IBD or animal models; ii) focused on CD8^+^ T cells from intestinal tissues or peripheral blood; iii) used scRNA-seq to analyze the mechanism of IBD; iv) discussed immunotherapies for IBD. The exclusion criteria were as follows: i) Unsuitable study designs, such as case reports, conference abstracts, comments or purely *in vitro* cell line studies; ii) data unsuitable for inclusion; iii) duplicate publications. A detailed description of the study selection process is shown in Fig. S1.

## 3. ScRNA-seq combined with other biological techniques to elucidate mechanisms

In IBD research, scRNA-seq integrated with complementary biological techniques has proven instrumental in delineating cell type-specific transcriptional heterogeneity (26,27). A multimodal study combining scRNA-seq with subcellular spatial transcriptomics (ST) has successfully tracked T cell populations associated with checkpoint inhibitor (CPI) colitis in intestinal tissues and identified distinctive features of mucosal IFN-γ-producing cytotoxic CD8^+^ T cells, thereby elucidating pathogenic pathways and informing novel preventive strategies (28). ScRNA-seq predicted that chemokines that are important for wound healing are altered during inflammation, in contrast to non-inflamed mucosa, with macrophages and CD8^+^ T cells in inflamed mucosa identified as key cellular targets (29).

Single-cell technologies have been utilized to profile distinct immune cell subsets in mucosal tissues and peripheral blood obtained from individuals with active or quiescent CD and UC, as well as healthy donors. Furthermore, when complemented with flow cytometry and RNA *in situ* hybridization validation, these methodologies offer substantial potential for developing precision therapies targeting distinct cellular subsets in IBD pathogenesis (30). ScRNA-seq revealed marked immune cell infiltration in 2,4,6-trinitrobenzene sulfonic acid (TNBS)-induced colitis and delineated the precise immune phenotypes, flow cytometry was subsequently employed to validate the dynamic changes in CD8^+^ IFN-γ^+^ cytotoxic T lymphocyte cells (CTLs) across disease and therapeutic states (31). To elucidate the role of IL-23 as a therapeutic target in chronic inflammation, an integrated approach combining single-cell transcriptomics and flow cytometry was applied to characterize IL-23 receptor-expressing cells in the gut, which identified group 3 ILC-intrinsic cytotoxic T-lymphocyte-associated antigen-4 (CTLA-4) as a key checkpoint restraining IL-23-driven pathology, with disruption of these lymphocytes shown to perpetuate chronic inflammation in IBD (32). Another study with a comparative analysis of colonic tissues demonstrated a marked enrichment of CTLA-4^+^ T cells in patients with CD compared to healthy controls, with a notable elevation in the rate of CD observed both in the CTLA-4^+^ subpopulations (33). These findings identify CTLA-4^+^ T cells as active contributors to intestinal inflammation. Through integrated scRNA-seq and mass cytometry analyses, Cao *et al* (34) demonstrated that Crohn's-like disease of the pouch involved a systemic remodeling of immune and stromal compartments in the pouch and prepouch ileum, characterized by clonally expanded effector CD4^+^ T and CD8^+^ T cell populations.

By integrating genome-wide association studies with scRNA-seq for credible causal mapping, the study identified monogenic and polygenic IBD genes highly expressed in phagocytes (circulating monocytes, neutrophils, DC2s and macrophages) and activated T cells (CD8^+^ IL-17^+^ and circulating T cells) which establishes a clinically actionable framework for classifying and managing monogenic IBD while delineating shared and distinct features between monogenic and polygenic forms (35). ScRNA-seq and RNA scope on terminal ileum biopsies validated upregulated NOD-like receptor family CARD domain containing 5 and transporter associated with antigen processing 1 in the intestinal epithelium of patients with CD, showing co-localization with CD8^+^ T cells, suggesting epithelial-lymphocyte crosstalk mediated by mediated by major histocompatibility complex class (MHC-I) (36).

The integration of scRNA-seq with complementary multiomics platforms and functional validation studies (26-28,30) has established a multidimensional analytical framework. This integrated approach not only systematically delineates the heterogeneity of CD8^+^ T cells, their microenvironmental crosstalk and clinical associations in IBD but, more importantly, establishes a causal relationship between cell states, underlying molecular mechanisms, functional phenotypes and clinical disease manifestations. This framework provides a novel conceptual and mechanistic dimension to the understanding of IBD immunopathology and directly informs the rationale for developing precision therapeutic strategies focused on CD8^+^ T cell biology modulation.

## 4. The role and heterogeneity of CD8^+^ T cells in IBD

Notably, CD8^+^ T cells in the colonic tissues of patients with IBD exhibit remarkable phenotypic and functional heterogeneity. In pediatric patients with IBD with active disease, colonic tissues exhibited clonal expansion of CD8^+^ TRM subsets and GZMK^+^ (granzyme K) CD8^+^ effector memory T cells. Patients with active IBD exhibited decreased frequencies of ITGAE^+^ and ENTPD1^+^ CD8^+^ TRM cells compared with inactive controls, alongside frequent interconversion between these subsets, indicative of a potential developmental relationship (37).

In UC, CD8^+^ TRM cells particularly demonstrated a pronounced inflammatory differentiation shift (19). Three main signals, antigen, co-stimulation and inflammation, regulate T cell proliferation and effector and memory CD8^+^ T cell differentiation (38). Under physiological conditions, TRM cells maintain a balanced differentiation state. In UC, TRM cells can upregulate eomesodermin (Eomes), which transcriptionally modulates a network of genes encompassing inflammatory cytokines (Ifng), cytolytic effectors (Gzma), chemokines (Ccl3-5), survival regulators (Bach2, Cd27 and Il2rb), killer cell lectin-like receptors (Klrb1, Klrc1, Klrd1, Klrg1 and and Krk1), co-stimulatory molecules (Tnfrsf18/GITR and Tnfrsf4/OX40) and cell-surface receptors (Crtam) (19,39). T-bet and Eomes, members of the T-box transcription factor family, are involved in the differentiation and functional programming of effector and memory CD8^+^ T cells. During early T cell activation, these factors cooperatively upregulate the expression of IFN-γ, granzyme B, perforin, CXCR3 and CXCR4, leveraging partially overlapping transcriptional functions to promote CTL development (40-42). CD8^+^ T cells lacking both T-bet and Eomes lose their CTL identity and aberrantly adopt an IL-17-producing phenotype (43). Single-cell profiling of UC colonic biopsies showed CD8^+^ IL-17^+^ T cells and Tregs progressively expanding along the health-to-inflammation axis, becoming primary IL-17 and TNF producers, with CD8^+^ IL-17^+^ T cells linked to pathogenic potential and tissue damage (44). Through integrated multi-omics analyses, Gupta *et al* (28) successfully tracked T cell populations associated with CPI-induced colitis in intestinal tissues, identifying characteristic cytotoxic CD8^+^ T cells signatures derived from TRM and peripheral circulating populations. Single-cell resolution analyses of UC revealed spatially specialized immune dysregulation mediated by immunometabolic mechanisms, demonstrating that human colonic lamina propria macrophages and CD8^+^ T cells demonstrate an effector-like phenotype characterized by enhanced activation status, while their lipid metabolism has been suppressed compared with colonic epithelium (45).

Multiple single-cell analyses of CD revealed altered heterogeneity and subset distribution of intestinal intraepithelial T cells, identifying unique intraepithelial lymphocytes (IELs) populations in the terminal ileum of patients with CD, including NKp30^+^ γδT cells that express RORγt and produce IL-26 upon NKp30 engagement (46). Comparative analyses of non-inflamed/inflamed CD tissues vs. healthy controls showed that expanded T-helper 17 cells but diminished CD8^+^ T, γδT, T_FH_ and Treg populations in inflamed tissues, while parallel lamina propria findings demonstrated increased CD8^+^ and decreased CD4^+^ T cells, with elevated Th17-to-Treg/T_FH_ ratios (46). Pseudotemporal trajectory analyses inferred from transcriptional profiles uncovered divergent differentiation trajectories among T cell subsets, with T cell populations in CD preferentially differentiating from Tregs toward Vδ2 γδ T cells, whereas in colorectal cancer, the dominant trajectory progressed from CD8^+^ T cells to Tregs (47). A previous study indicated that CEACAM5-stimulated CD8^+^ T cells markedly inhibit the proliferation of CD4^+^ T cells *in vitro*, implicating their function as key modulators in maintaining intestinal immune homeostasis (48).

In IBD, CD8^+^ T cells exhibit high heterogeneity and functional plasticity (10,17,18). UC was characterized by the inflammatory reprogramming of colonic CD8^+^ TRM, which upregulate Eomes and produce pro-inflammatory cytokines and cytotoxic mediators, alongside the emergence of CD8^+^ IL-17^+^ T cells with tissue-damaging potential (43,44). In CD, a reduction in intraepithelial CD8^+^ T cells contrasts with an expansion in the lamina propria, accompanied by distinct γδT cell subsets (46,47). Both subtypes exhibit clonally expanded CD8^+^ TRM and effector memory populations, as well as dynamic interconversion between TRM subsets (37). Through cytokine secretion, direct cytotoxicity and immunoregulatory functions, such as suppressing CD4^+^ T cell proliferation, CD8^+^ T cells critically contribute to IBD immunopathology. Their functional states associate with disease activity, tissue inflammation and metabolic dysregulation, highlighting specific subsets as potential targets for precision immunotherapy.

## 5. CD8^+^ T cell subsets in IBD: Signaling and clinical relevance

The widespread application of single-cell sequencing analyses has identified the heterogeneity of CD8^+^ T cell infiltration in numerous human diseases, including malignancies such as non-small cell lung cancer (49), breast cancer (50), head and neck cancer (51), liver cancer (52), melanoma (53), bladder cancer (54) and colorectal cancer (55), as well as immune-related diseases such as IBD (56,57) and COVID-19 (58). Research on CD8^+^ T cells in IBD, reveal that these cells are primarily categorized into naïve, cytotoxic, regulatory, proliferative, memory and exhausted subtypes (Table I). Corridoni *et al* (56) utilized single-cell transcriptomics combined with T cell receptor repertoire analyses and flow cytometry to establish a comprehensive map of CD8^+^ T cells in healthy and UC colonic tissues, classifying CD8^+^ T cells into 14 subgroups, including naïve, memory, effector, TRM, double-positive CD8^+^ CD4^+^ cells, mucosal-associated invariant T cells, IELs and TF-related T cells like IL-26^+^-expressing CD8^+^ T cells.

CD8^+^ T cells exhibiting elevated expression of SELL, CCR7, LEF1 and TCF7 are transcriptionally defined as naïve cells (59). T cells originate from bone marrow-derived progenitor cells that undergo differentiation in the thymus and are principally categorized into CD4^+^ or CD8^+^ αβ T cell lineages, which recognize peptide antigens presented by MHC molecules on antigen-presenting cells. After encountering antigens, naïve CD8^+^ T cells undergo activation, clonal expansion and differentiation into effector and memory subsets, acquiring the capacity to secrete cytokines, modulate immune responses and mediate cytotoxicity (60). Oxidative phosphorylation is the primary metabolic pathway in resting naïve CD8^+^ T cells. Activated CD8^+^ T cells initiated broad transcriptional reprogramming, shifting their core metabolic program toward aerobic glycolysis while enhancing protein and lipid biosynthesis and markedly enlarging cell size to provide sufficient materials and energy for subsequent large-scale proliferation (61,62).

Long-lived CD8^+^ T cells exhibit notable heterogeneity and are typically classified into TRM cells, effector memory cells and central memory cells, which differ in anatomical distribution, recall capacity and effector functions, collectively establishing a multi-layered defense system against recurrent infections (63). TRM cells, which persistently localize in mucosal barriers such as the lung, skin and reproductive tract and exhibit high CD103/CD69 expression, function as non-recirculating sentinel cells that provide rapid tissue-specific immune protection through direct effector activity and localized reactivation of immune responses (64). In UC, clonally expanded CD8^+^ TRM cells undergo a pronounced transition toward a pro-inflammatory phenotype, partly driven by the upregulation of Eomes, which reinforces their pathogenic features through enhanced inflammatory cytokine production and cytolytic activity (19). Clonal CD8^+^ T cell expansion is predominantly observed within CD103^+^ (ITGAE^+^) and CD39^+^ (ENTPD1^+^) TRM subsets, as well as in GZMK^+^ effector memory T cells, with dynamic inter-conversion between these populations indicating a potential developmental continuum (37). Compared with TRM cells, cytotoxic CD8^+^ GZMB^+^ cells express higher levels of cytolytic genes, effector molecules and chemokines, aligning with their role as effector cells causing terminal epithelial damage (65). During tumor progression, the majority of new clones in the bloodstream similarly expressed cytotoxic markers such as GZMH, GZMB, KLRD1 and FGFBP2; however, failed to inhibit tumor growth, if targeting tumor antigens, these markers may also be modified and amplified (66). Nagahara *et al* (67) found that, compared with the CD8^+^ ITGAE^+^, the exhibition of CD8^+^ ENTPD1^+^T cells increased antioxidant gene expression, implicating oxidative stress in UC pathogenesis (68); hence, it is hypothesized that CD8^+^ ENTPD1^+^T cells may provide additional protection by maintaining redox homeostasis in the colon (37).

CD8^+^ T cells with different phenotypes carry out distinct roles in IBD. A single-cell atlas of CD8^+^ T cells in UC revealed innate-like properties in CD8^+^ IL-26^+^ T cells (56), whereas separate investigations documented a reduction in cytotoxic IFN-γ^+^ CD45RA^+^ effector memory CD8^+^ T cells within the mucosal compartment in UC (30). CTLs are characterized by their ability to mediate killing through the secretion of cytokines and cytolytic molecules which are transferred to target cells (69). X-box binding protein 1 (XBP1), a central regulator of endoplasmic reticulum stress, modulates glucose and lipid metabolic pathways to fulfill bioenergetic and biosynthetic requirements, thereby supplying essential metabolic intermediates that support CD8^+^ T cell activation and clonal expansion (70). The IRE1α-XBP1 signaling axis facilitates the production and release of effector molecules, including KLRG1, granzyme B and perforin, thereby enhancing the cytolytic capacity of CTLs (71); however, CD8^+^ tumor-infiltrating lymphocytes (TILs) demonstrate upregulated XBP1 expression, which drives the transcriptional induction of multiple inhibitory receptors and contributes to T cell exhaustion (72). Simultaneously, PERK pathway activation in CD8^+^ TILs induces CHOP and ATF4, which repress T-bet expression and, thereby, impair antitumor function, whereas genetic deletion of PERK or CHOP enhances their activation, expansion, effector activity and longevity (73).

Tyrobp, an established modulator of pro-inflammatory mediator synthesis in macrophages and neutrophils, and has been mechanistically associated with the development of multiple inflammation-associated disorders (74). Genetic ablation of Tyrobp markedly attenuates the severity of dextran sulfate sodium (DSS)-induced colitis in murine models (75), suggesting it may serve as an upstream regulatory molecule in UC (76). Rosati *et al* (77) performed comprehensive TCRα and β chain repertoire analyses using high-throughput sequencing on peripheral blood and intestinal tissues from patients with IBD and healthy controls. Subsequent scRNA-seq identified distinct T cell clonotypes, including a CD-associated invariant T cell, enriched within the CD8^+^ effector memory CD161^+^ subset and marked by elevated expression of KLRD1 (CD94) and KLRB1 (CD161), molecules indicative of innate-like and NK cell-like phenotypes (77). Lymphocyte activation gene-3 (LAG-3) is an immune checkpoint that identifies activated lymphocytes and may contribute to inflammation (78). Elevated frequencies of LAG3^+^ cells, particularly within activated effector memory T cell populations in the inflamed mucosa, associate positively with disease activity in UC and genetic ablation of LAG-3 depletes proliferating T cells, supporting its potential as a therapeutic target (78).

This study by Dai *et al* (79) investigated diagnostic stratification and treatment response prediction in CD through T cell marker gene expression, single-cell transcriptomes were acquired from both CD and normal specimens, after which cell subtype annotation and intercellular communication analyses were performed. This analysis identified key genes that are positively associated with the majority of immune genes, particularly immune checkpoint-related genes such as *CTLA4*, *CD86*, *PDCD1LG2* and *CD40* (79). The study found that only a subset of CD8^+^ T cells in patients with CD with high activity were highly activated and expressed exhaustion markers, potentially representing CD-reactive cells (57). The cytokine profiles of these CD39 and PD-1 CD8^+^ T cells were evaluated as surrogates for effector function and were associated with the clinical course, exhibiting the typical functional cytokine pattern of exhausted T cells and associating with milder disease (57). Additionally, Huang *et al* (80), using multi-omics approaches, identified unique Th17-like cells, exhausted Tc17 cells and proliferation-associated CD8^+^ T cell subsets in patients with UC.

**Figure 2:**
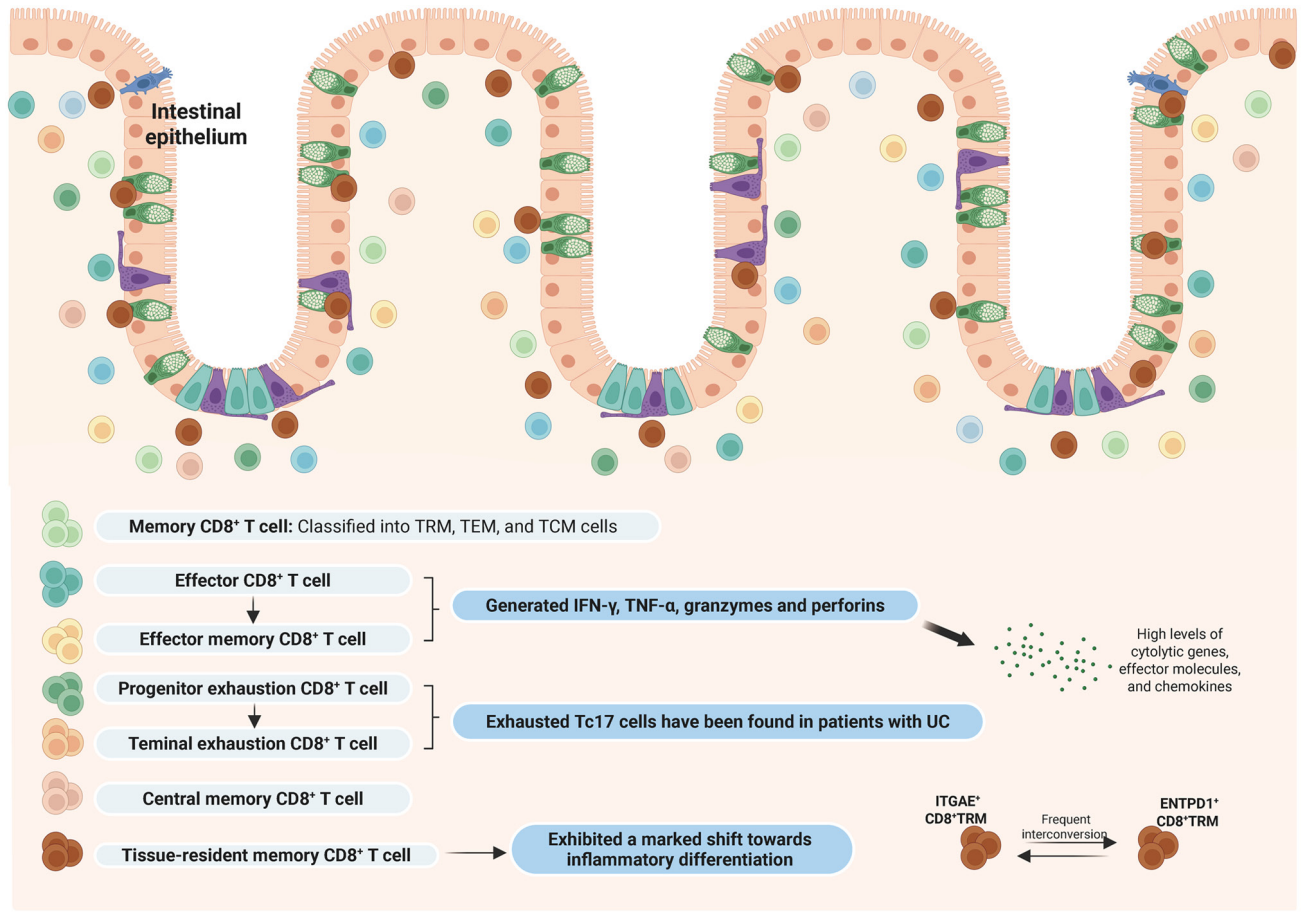
Different CD8^+^ T cell subsets in IBD. The function of CD8^+^ T cells in IBD is complex and multifaceted, with various subtypes contributing differently to disease pathogenesis and progression. Advanced single-cell methodologies have enabled high-resolution dissection of cellular heterogeneity and functional specialization within the immune compartment, revealing novel candidate therapeutic targets for IBD intervention. IBD, inflammatory bowel disease; TRM, tissue-resident memory T cells; TEM, effector memory T cells; TCM, central memory T cells. The figure was prepared using Biorender.com (agreement no. WO29A0XD5G).

The function of CD8^+^ T cells in IBD is complex and multifaceted, with various subtypes contributing differently to disease pathogenesis and progression. Advanced single-cell methodologies have enabled high-resolution dissection of cellular heterogeneity and functional specialization within the immune compartment, revealing novel candidate therapeutic targets for IBD intervention. Further research is necessary to fully elucidate the mechanisms by which these cells influence inflammation and to develop targeted therapies that can modulate their activity and function in IBD (Fig. 2).

## 6. Cross-talk between CD8^+^ T cells and other immune or non-immune cells

**Figure 3:**
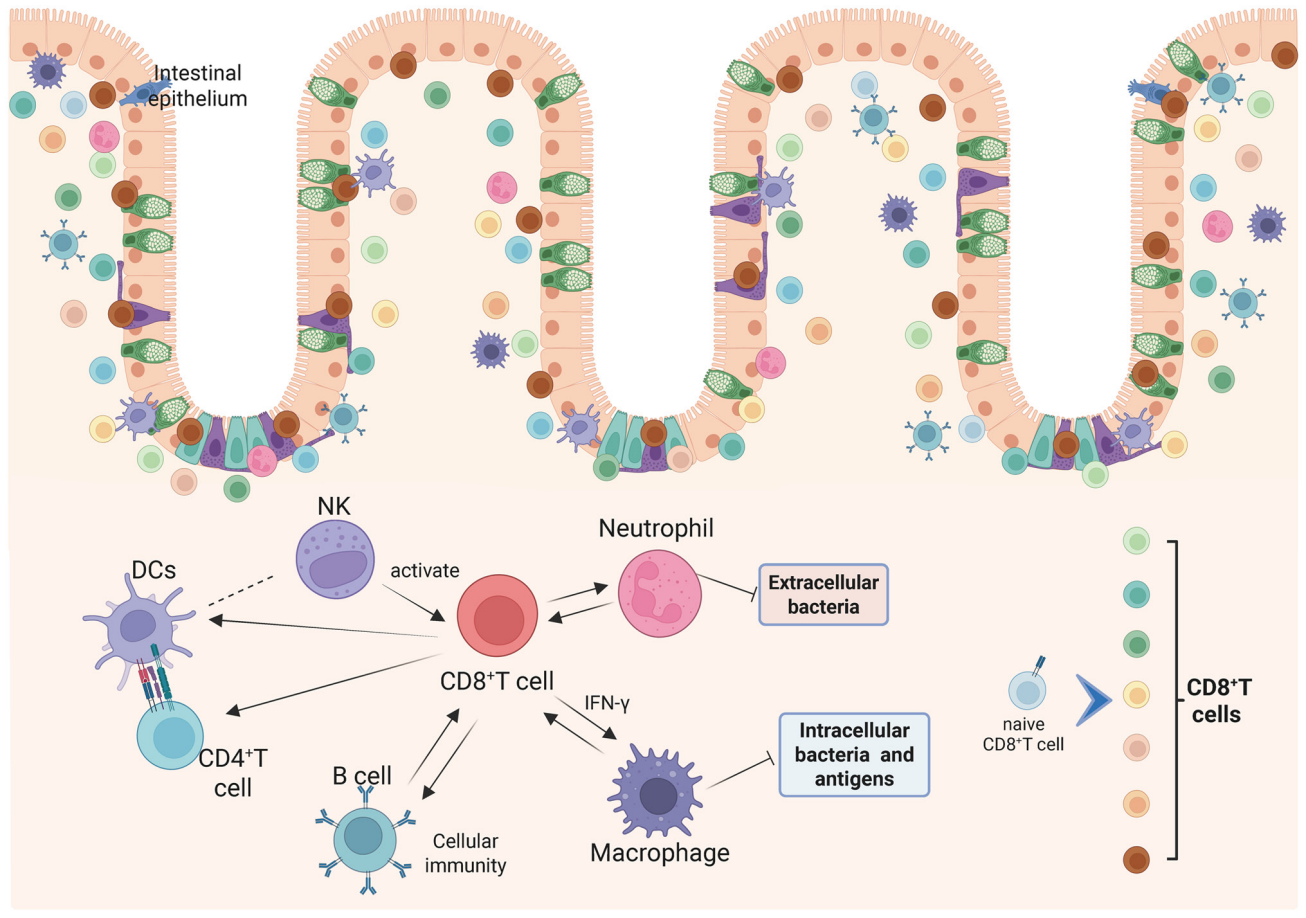
The interaction between CD8^+^ T cell and other immune cells. Immune responses are mediated by various cell types, including classical immune cells such as macrophages, NKs and T cells. CD8^+^ T cells are associated with activated DCs, macrophages, neutrophils, NK cells, B cells and CD4^+^ T cells. NK, natural killer; DCs, dendritic cells. The figure was prepared using Biorender.com (agreement no. SH29A0XYQM).

Immune responses are coordinated by diverse cellular participants, encompassing classical immunocytes, such as macrophages, natural killer cells and T lymphocytes, as well as structural cells including epithelial, endothelial and stromal elements (81). Several studies have focused on changes in immune cell composition during UC, revealing increased infiltration levels of CD8^+^ T cells, Tregs, macrophages, DCs, neutrophils and CD4^+^ T cells in patients with UC compared with healthy individuals (44,82-84). To investigate immune infiltration patterns in UC, Huang *et al* (85) acquired three transcriptional datasets from the GEO database, identified differentially expressed genes via linear models for micro-array data, and conducted functional enrichment analyses to delineate associated biological processes. A study comprising a correlation analyses of immune cells indicated that CD8^+^ T cells, apart from being unrelated to immature DCs, showed correlations with activated DCs, macrophages, neutrophils, NK cells, B cells, Th1, Th2, TILs and Tregs (85) (Fig. 3).

As a pivotal T cell subset, CD8^+^ T cells have been observed to exhibit reduced frequencies in patients with CD (86), aligning with earlier reports of impaired CD8^+^ T cell reactivity to commensal microbiota in IBD (87). Given the key role of DC-mediated antigen presentation in the initiation of CD8^+^ T cell responses, the elevated frequency of functionally impaired DCs observed in the present review may underlie the compromised CD8^+^ T cell reactivity in this context. Another study demonstrated that nucleotide-binding oligomerisation domain-containing protein 2 (NOD2), via the cross-presentation pathway, facilitated CD8^+^ T cell activation, but CD-associated NOD2 polymorphisms impaired this activation process (88).

Patients with active CD exhibit notably elevated immune scores compared with those with inactive disease, characterized by a reduced abundance of CD8^+^ T cells, alongside increased infiltration of M0 and M1 macrophages and neutrophils, indicating distinct immune cell profiling between disease states (89). Correlation analyses of different immune cells in patients with CD showed a negative correlation between CD8^+^ T cells and immune scores, M0 and M1 macrophages, and neutrophils, whereas there is a positive correlation between immune scores, M0 and M1 macrophages, and neutrophils (89). Uncontrolled neutrophil accumulation in the intestinal lumen was associated with IBD pathogenesis. Bone marrow-derived neutrophils traversed the epithelium into the mucosal epithelium and subsequently entered the intestinal lumen, forming an important defensive barrier that can clear extracellular microorganisms (90). Neutrophils assisted in the recruitment of other immune cells and promoted mucosal healing by releasing anti-inflammatory factors, thereby attenuating the inflammatory response (91). Conversely, excessive neutrophils can exacerbate inflammation by compromising the integrity of the intestinal epithelial barrier, causing physical damage to the epithelium and secreting pro-inflammatory cytokines (92).

NK group 2 member D (NKG2D) is one of the most characteristic receptors shared by NK cells and T cells (93). This molecule contributes to IBD pathogenesis via its expression on intestinal cytotoxic lymphocytes and upregulated ligand presentation within inflamed mucosal tissues (94,95). NKG2D inhibition enhanced the migratory capacity and expansion of CD8^+^T cells in co-culture systems with NKG2D-ligand-expressing astrocytes, underscoring its functional involvement in CD8^+^ T cell-astrocyte crosstalk (96). Evidence indicated that invariant NKT (iNKT) cells and Tregs suppressed DSS-induced colitis, whereas disease progression was primarily driven by NK1.1^+^ CD8^+^ T cells, with CD1d-restricted iNKT cells and CD1d-independent NK1.1^+^ CD8^+^ T cells interacting to disrupt IFN-γ homeostasis and promoted intestinal inflammation, identifying NK1.1^+^ CD8^+^ T cells as potential therapeutic targets in human IBD (97).

The study exploring the TRM cell populations in colonic epithelial and lamina propria tissues adjacent to mucosa-associated microbes indicated that the fundamental pathology of human IBD involved a reduced response of CD8^+^ T cells to commensal bacteria, leading to a deficiency of TRM cells in the colon, and this was associated with chronic B cell activation and excessive IgA secretion, linked to a loss of barrier immunity (87). In chronic DSS-induced colitis, pronounced B cell and modest CD8^+^ T cell infiltration into enteric ganglia, unlike minimal infiltration in acute DSS, TNBS or metastatic colitis, may drive visceral pain and neuronal apoptosis, contributing to hypersensitivity in IBD (98). IL-35 exerted anti-inflammatory activity by enhancing regulatory B cell function, which suppressed proliferation and the production of cytokines, including IFN-γ, IL-17 and TNF-α, by autologous CD4^+^ CD25^−^ T cells and CD8^+^ T cells, however, this immunoregulatory mechanism was impaired in UC (99).

CD4^+^ and CD8^+^ T lymphocytes contribute to immune dysregulation in IBD through the secretion of proinflammatory cytokines, orchestration of innate and adaptive immune responses and direct cytopathic effects on intestinal epithelial and stromal components (100-102). Within T cell subsets, TRM precursor cells and cytotoxic effector cell populations are closely associated with CPI colitis. CD4^+^ and CD8^+^ TRM populations, along with CD8^+^ GZMB^+^ cytotoxic populations, were markedly enriched in colonic tissues of patients with immune CPI colitis. This accumulation suggested that CD4^+^ and CD8^+^ TRM cells served as tissue-localized precursors which, upon antigen encounter, give rise to CD8^+^ GZMB^+^ progeny contributing to epithelial injury in immune CPI colitis (65).

Before the onset of recurrent colitis, interferon-producing cytotoxic CD8^+^ T cells were generated in the mesenteric lymph nodes against hapten-modified self-proteins. Following toxin exposure, CD8^+^ T cells were rapidly recruited to the colonic lamina propria and mediated cytotoxicity against hapten-modified epithelial cells via granzyme B expression (101). TNBS exposure induced the generation of haptenated self-antigens within the colon, comprising modified host tissue proteins and bacterial constituents (103), leading to the activation of CD8^+^ CTL effector molecules that cross-react with autologous proteins (104). Upon hapten challenge, these CD8^+^ effectors were recruited to the colon, initiating inflammation by lysing epithelial cells (101). Under stimulation, CD8^+^ cytotoxic IELs rapidly secreted IFN-γ, enhancing the cytotoxic response of T cells against infected epithelial cells and increasing barrier permeability (105-107). Furthermore, CD8^+^ T cells within the intestinal mucosa exhibit elevated expression of natural killer receptors, including NKG2D and KLRD1, which facilitated the recognition of stress-induced ligands on epithelial cells (108). These findings highlighted the important role of CD8α^+^ T cells and TGF-β-independent Smad4 signaling in regulating the accumulation, spatial distribution and pathogenicity of CD8αβ^+^ T cells in the intestinal microenvironment, thereby mitigating severe chronic inflammation (109). CD8αβ^+^ T cells, rather than CD4^+^ T cells, are predominantly located in the lamina propria, which may allow CD8^+^ T cells to initiate epithelial damage in a microbiota-dependent manner, leading to subsequent inflammation (109). In various IBD models, CD8^+^ T cells were implicated in the killing of IECs, thereby initiating chronic inflammation through different mechanisms (101,110). Elevated IFN-γ expression in CD8^+^ T cells adjacent to colonic epithelium has been documented in IBD (111), where it promoted the disruption of the intestinal mucosal barrier and augmented proinflammatory responses, thereby exacerbating disease pathogenesis (112,113).

## 7. Immune therapies for IBD and their challenges

**Figure 4:**
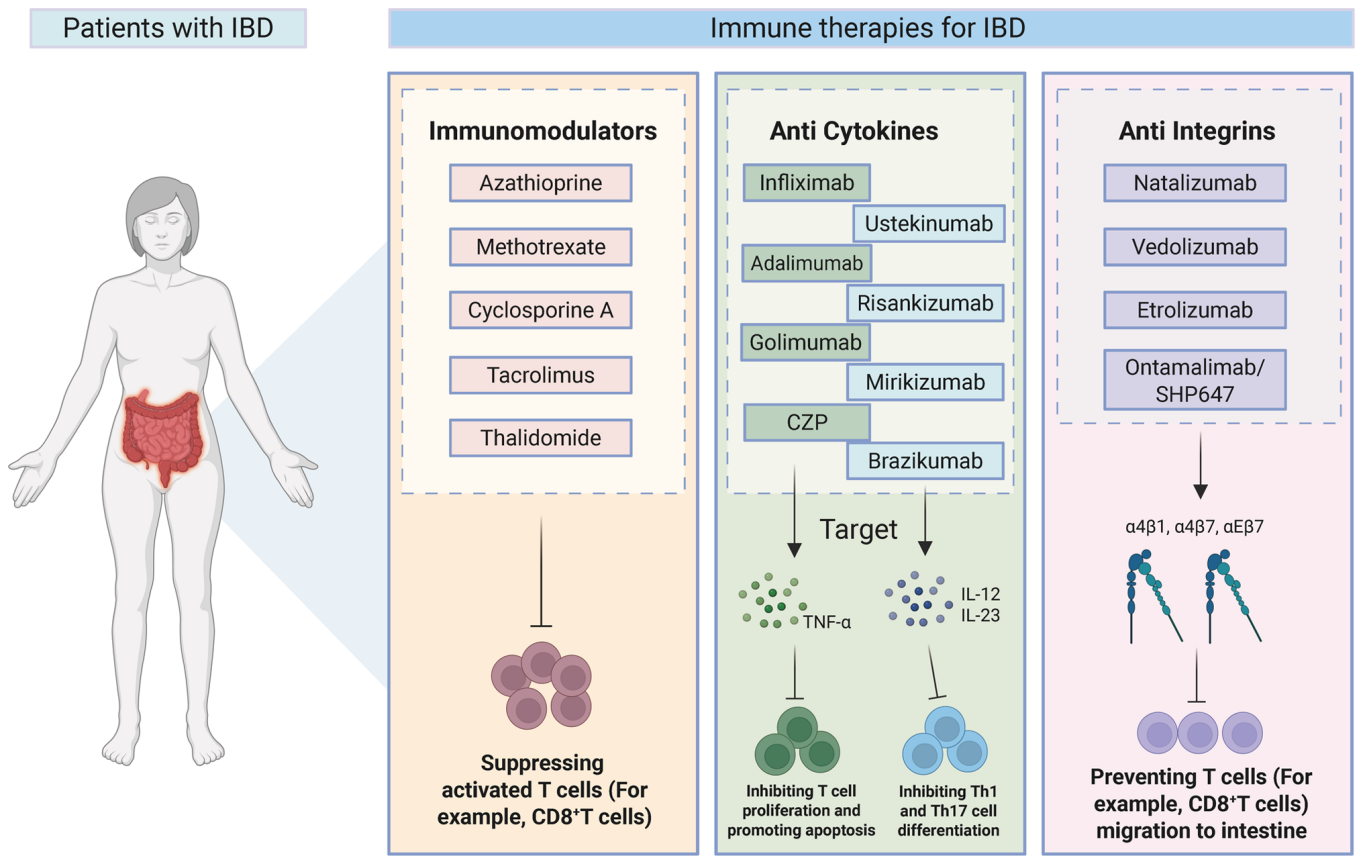
The immune therapies for IBD. Immunomodulators and biologics are currently widely utilized as common therapeutic agents for the treatment of IBD. Azathioprine, methotrexate, cyclosporine A, tacrolimus and thalidomide are commonly used as immunomodulators by suppressing activated T cells such as CD8^+^ T cells. Infliximab, adalimumab, golimumab and CZP are approved as anti-TNF drugs for IBD. The intestinal mucosa of patients who do not respond to infliximab exhibited increased effector CD8^+^ T cells alongside a reduction in cytotoxic CD8^+^ T cells. Ustekinumab, risankizumab, mirikizumab and brazikumab target IL-12 and IL-23. Natalizumab, vedolizumab, Etrolizumab and Ontamalimab/SHP647 prevent the migration of T cells such as CD8^+^ T cells to the intestine by targeting α4β1, α4β7/or αEβ7 integrins. CZP, certolizumab pegol. The figure was prepared using Biocender.com (agreement no. WK29A0Y58I).

IBD is an intestinal immune disorder caused by abnormal immune responses to environmental changes in genetically predisposed individuals, where innate immunity and adaptive immunity both play key roles in its onset and progression (6). Research has indicated that immune regulatory dysfunction may represent the intrinsic core of IBD pathogenesis, characterized by chronic intestinal inflammation and tissue damage resulting from aberrant expression of pro-inflammatory and anti-inflammatory factors in both innate and adaptive immunity (114). The medical treatment of IBD primarily encompasses 5-aminosalicylic acid (5-ASA), glucocorticoids, immunomodulators, biologics, antibiotics and stem cell transplantation, with ongoing advancements in immune-related drug research and development driven by in-depth studies on IBD pathogenesis (115,116). Immunomodulators and biologics are currently widely utilized as common therapeutic agents for the treatment of IBD (115,116) (Fig. 4).

Azathioprine (AZA), methotrexate (MTX), cyclosporine A (CsA), tacrolimus and thalidomide are commonly used immunomodulators in IBD (117,118). The active end-product of AZA, 6-thioguanine, incorporates into the DNA and RNA of T cells, exerting inhibitory effects on cell activity and cytotoxicity; however, due to its slow induction, AZA is recommended for use in combination with TNF inhibitors (119). The 2023 European Crohn's and Colitis Organisation (ECCO) guidelines recommend AZA monotherapy for maintaining remission in steroid-dependent UC or in patients intolerant to 5-ASA (120). Randomized controlled trials have demonstrated that AZA was superior to placebo in maintaining clinical remission (121,122). Low-dose MTX, while exhibiting anti-inflammatory properties, is ineffective as a monotherapy for inducing remission in CD or UC (123), and it is typically combined with TNF inhibitors to achieve therapeutic efficacy (124). To the best of our knowledge, there is no evidence supporting the use of MTX monotherapy for maintaining remission in UC (125). A clinical study demonstrated that MTX showed no superiority over placebo in achieving steroid-free clinical remission (126). CsA and tacrolimus, both calcineurin inhibitors, effectively suppress activated T cells and demonstrate therapeutic efficacy in UC, although their long-term use requires monitoring due to potential toxic side effects (127,128). Thalidomide can serve as an alternative treatment for pediatric refractory CD in cases of secondary failure to anti-TNF therapy (129); however, its widespread use in IBD is limited by adverse effects such as fatigue, somnolence, peripheral neuropathy and the risk of long-term complications including deep vein thrombosis and acute pancreatitis (130). According to the 2023 ECCO guidelines, CsA, tacrolimus and thalidomide were not recommended as conventional or first-line therapies for either UC or CD (120).

TNF-α, an important pro-inflammatory cytokine, stimulates T cells (131,132), recruits inflammatory cells to inflammatory tissues and involved in IBD progression. Anti-TNF-α therapy exerts its effects through multiple mechanisms, such as neutralizing TNF-α, reverse signaling, apoptosis and cytotoxicity (133). Infliximab (IFX), adalimumab (ADA), golimumab (GLM) and certolizumab pegol (CZP) are approved by the Food and Drug Administration and European Medicines Agency (EMA) as anti-TNF drugs for IBD treatment. IFX, ADA and CZP all have similar ability to neutralize TNF-α and prevent the transmission of anti-inflammatory signals by inhibiting the binding of TNF-α to its receptors (133,134). Reverse signaling occurs when anti-TNF-α agonists engage transmembrane TNF-α, initiating intracellular signaling cascades in cells expressing the ligand. Mitoma *et al* (135) demonstrated that IFX triggers a potent reverse signal capable of suppressing T cell proliferation by inducing cell cycle arrest at the G0/G1 phase. In addition, IFX increases apoptosis of T lymphocytes in patients with CD (132,136). IFX, ADA and GLM were recommended for inducing remission in patients with moderate-to-severe UC who have had an inadequate response to, or were intolerant to, conventional therapy in the 2023 ECCO guidelines (120). Although studies that entail direct comparisons with anti-TNF agents are unavailable, several meta-analyses have demonstrated that IFX was superior to both ADA and GLM in inducing clinical remission or response (137,138). The combination of IFX with AZA was more effective than IFX monotherapy (139). Furthermore, according to a recent study, an elevated proportion of CCL5^+^ effector CD8^+^ T cells associated with disease activity, lesion expansion and IFX resistance (140). Specifically, the intestinal mucosa of patients who do not respond to IFX exhibited increased effector CD8^+^ T cells alongside a reduction in cytotoxic CD8^+^ T cells and NK cells (140).

Ustekinumab (UST) selectively binds the common p40 subunit of IL-12 and IL-23, thereby suppressing Th1 and Th17 cell differentiation and modulating T follicular helper cell development, contributing to its efficacy in the acute and chronic phases of inflammatory disease (141-143). UST exhibits a rapid onset of action, considerably improving patient symptoms ≤7 days, with the subcutaneous route of administration demonstrating relatively superior efficacy (144). This medication has been approved for the treatment of CD and UC. The 2023 ECCO guidelines recommended UST for the treatment of patients with moderate-to-severe active UC who have an inadequate response to, or are intolerant to, conventional therapy. A clinical study demonstrated that UST provided benefit over placebo in both inducing and maintaining clinical remission, improving clinical response and ameliorating endoscopic scores in patients with moderate-to-severe UC (145). Risankizumab (RIS), mirikizumab and brazikumab, all targeting the IL-23 p19 subunit, exhibit robust clinical efficacy in the induction and maintenance of remission in IBD, accompanied by favorable tolerability and safety profiles (146-148).

Integrins, adhesion molecules and signaling proteins on the leukocytes' surface are activated through intracellular signaling triggered by chemokines and other stimuli, enabling leukocytes to migrate across the vascular wall and infiltrate tissues such as the intestine, so antagonizing integrins can block the binding of lymphocytes to adhesion molecules and their migration into the gut, thereby alleviating local inflammatory responses (118,149). Natalizumab, which effectively targets α4β1 and α4β7 integrins, selectively inhibiting lymphocyte migration to inflammatory sites by blocking α4 integrin signaling, was the first drug approved for the treatment of CD (150). α4β7 expression facilitates the infiltration of regulatory T cells into intestinal tissue, while its inhibition diminishes the intestinal homing of regulatory and effector T cells (151). Adoptive transfer of α4-deficient T cells into immunodeficient mice compromised their trafficking to inflammatory foci, leading to a marked attenuation of chronic colitis severity (152). Compared with natalizumab, vedolizumab specifically targets α4β7, selectively inhibiting lymphocyte trafficking to the gut while exhibiting fewer systemic adverse effects (153). The GEMINI I phase 3 induction study demonstrated that a higher proportion of patients treated with vedolizumab achieved endoscopic remission compared with those receiving placebo (154). Therefore, it is also recommended by the ECCO guidelines for inducing remission in patients with moderate-to-severe active UC (120). Etrolizumab blocks cell adhesion by targeting α4β7 and αEβ7 integrins (155), demonstrating considerable efficacy in UC and CD, with favorable safety profiles for subcutaneous administration. Ontamalimab/SHP647 binds to mucosal addressin cell adhesion molecule (MAdCAM), preventing lymphocyte migration to the gut and, thereby, attenuating inflammation, and has shown potential for long-term maintenance of remission in UC with a favorable safety profile (156).

With the continuous advancement of clinical and basic research, the limitations of conventional pharmacological therapies have become increasingly apparent and the development of biosimilars and small-molecule drugs has been addressing the shortcomings of previous treatments in previous years (115). Researchers may leverage multi-omics technologies to identify potential biomarkers and explore novel immunotherapeutic targets and drugs, while integrating disciplines such as immunology and microbiology to drive innovation and advancement in IBD immune-based therapies.

## 8. Challenges and future directions in understanding CD8^+^ T cells

The integrated colonic barrier system is composed of four essential elements: An immune barrier mediated by innate and adaptive immune cells, a mechanical barrier formed by IECs and tight junctions, a biological barrier dominated by the gut microbiota, and a chemical barrier comprising the mucus layer (for example, MUC proteins) and digestive enzymes (157). CD8^+^ T cells, as essential immune cells, participate in the pathological damage of IBD. However, their subpopulation composition, the signaling pathways they influence, their interactions with other colonic cells and how they dynamically remodel to impact inflammation in IBD remain unclear. This could be a viable direction for future research. Despite the various classifications of CD8^+^ T cell subpopulations in IBD, considerable heterogeneity exists among studies, and there is no unified definition or explanation of their roles, which poses a challenge for readers. The descriptions in the present review are relatively simple, and there is hope for more standardized research in the future.

The colonic environment exhibits high heterogeneity, and CD8^+^ T cells display notable diversity in phenotype and function. This complexity poses challenges in defining the specific interactions between CD8^+^ T cells and other colonic cells. Single-cell technologies such as scRNA-seq, offer valuable biological insights but are inherently limited by their resolution and sensitivity. They may miss rare cell populations or subtle transcriptional changes that are important for understanding CD8^+^ T cell interactions. Interactions between CD8^+^ T cells and other colonic cellular constituents are highly dynamic and context-dependent, modulated by inflammatory signals, infectious stimuli and tissue injury. Capturing these transient interactions in static snapshots remains a notable challenge. Translating basic findings on CD8^+^ T cell interactions into clinical practice remains challenging. A deeper mechanistic understanding of their roles in disease progression and therapeutic response is essential to advance translational research in IBD.

ScRNA-seq has unveiled novel insights into the functional diversity and pathogenic mechanisms of CD8^+^ T cells, offering promising avenues for elucidating their roles in immunity and disease. First, the continued development and refinement of single-cell technologies, particularly multiomics approaches, will provide a more comprehensive understanding of CD8^+^ T cell interactions with higher resolution and depth. Furthermore, the integration of scRNA-seq with advanced *in vivo* imaging techniques, such as intravital microscopy, will allow for the visualization and real-time analysis of CD8^+^ T cell dynamics within their native tissue microenvironment, offering a clearer understanding of their spatial and temporal interactions with other colonic cells, particularly in the context of IBD. Furthermore, combining single-cell data with ST, proteomics and metabolomics will provide a holistic view of cellular and molecular interactions in the colon, elucidating the complex interplay between CD8^+^ T cells and the colonic microenvironment. In addition, conducting longitudinal studies to track changes in CD8^+^ T cell interactions over time, particularly during disease progression or therapeutic interventions, will reveal their dynamic roles and regulatory mechanisms, offering important insights into disease outcomes and treatment responses. Finally, incorporating patient-derived samples and organoid models will strengthen translational relevance by closing the gap between fundamental research and clinical practice, thereby accelerating the development of targeted therapies and personalized treatment approaches for IBD and related immune-mediated disorders. Together, these advancements will deepen the understanding of CD8^+^ T cell biology and pave the way for innovative diagnostic and therapeutic approaches.

Although current therapeutics of IBD are not specifically designed to target CD8^+^ T cells, emerging evidence suggests their potential to modulate this cellular subset. Advancements in scRNA-seq and complementary biological technologies have profoundly enhanced the resolution of CD8^+^ T cell heterogeneity and the adaptive dynamics of these cells in the inflamed intestinal niche. Future CD8^+^ T cells-targeted therapies could focus on three key strategies: i) Modulation of TRM by targeting surface markers such as CD103/CD69 to regulate their activation and homeostasis; ii) development of precision inhibitors against cytotoxic effector molecules for example, granzyme K, perforin or proinflammatory cytokines (IFN-γ/TNF-α); iii) induction of CD8^+^ regulatory T cell expansion and function via IL-2/IL-15 or TGF-β signaling pathways.

## 9. Conclusion

This review summarizes the research advances of single-cell transcriptomics in IBD over the past decade to unravel the complex heterogeneity and functional dynamics of CD8^+^ T cells, revealing their diverse phenotypes, interactions with immune and non-immune cells, and pivotal roles in inflammation and tissue homeostasis. The present review synthesizes previous findings and highlights the therapeutic potential of targeting specific CD8^+^ T cell subsets and their microenvironmental interactions, offering new avenues for precision medicine in IBD.

## Supplementary Data



## Figures and Tables

**Table I:** Functions and signaling pathways of CD8^+^ T cell subsets in IBD.

Cell populations	Clusters	Cell signaling/clinical relevance
Naïve	Naïve/CCR7^+^/SELL	The cell populations display a highly heterogeneous clonal architecture, with the majority of cells expressing unique TCR CDR3 sequence pairs (56).
Resident	T_RM_, ITGAE, ENTPD1, ZNF683	In UC, CD8^+^ TRM cells exhibit a marked shift towards an inflammatory differentiation state, which is associated with increased expression of the T-box transcription factor Eomes (19), clonal proliferation predominantly occurs within the CD8^+^ TRM subpopulations expressing ITGAE (CD8-ITGAE and CD8-ENTPD1) (37).
Cytotoxicity	GZMK^+^, GZMB^+^, IFNG	Clonal proliferation occurs in GZMK^+^ effector memory CD8^+^ T cells (37), cytotoxic CD8^+^ GZMB^+^ cells express higher levels of cytolytic genes, effector molecules and chemokines (65).
Cytokines	IL26^+^, IL17^+^, TYROBP^+^/ TYROBP^−^IELs	The expression of IL26 in colonic tissues is associated with the degree of inflammation, exhibiting characteristics of hybrid Tc17 and type 2 ILC3 (56), CD8^+^ IL-17^+^ T cells expand from healthy tissues to non-inflamed tissues and further to inflamed tissues, becoming the primary source of IL-17 (44), TYROBP^+/−^ cells share a substantial number of clones, facilitating the natural induction of IEL transformation (56).
Effector	FGFBP2^+^, CD161^+^, LAG3^+^	During the progression of various tumor diseases, new clones emerging in the blood or tumor tissues are enriched with cytotoxicity-related genes such as FGFBP2, which can be used as substitutes for cell or cytokine therapies (66), LAG3^+^ cells increase in inflamed mucosa, predominantly among effector memory T cells with an activated phenotype, potentially contributing to UC inflammation (76).
Immune checkpoints	CTLA4, PDCD1	Key genes in patients with CD or heathy samples show positive associations with most immune genes, particularly those related to immune checkpoints (79).
Proliferation-related	Proliferating	CD4^−^ Proliferating cells were found in T cell subpopulations with healthy donors (80).
Exhaustion-related	Exhausted Tc17	Discovering unique Th17-like cells and exhausted Tc17 cells in individuals with UC (80).

IBD, inflammatory bowel disease; TCR, T cell antigen receptor; UC, ulcerative colitis; TRM, tissue-resident memory T cells; CD, Crohn's disease.

## Data Availability

Not applicable.
