# Supplementary figures and images for: The roles and heterogeneity of CD8+ T cells in inflammatory bowel disease: A narrative review of insights from single-cell transcriptomics (Review)
Source: Int J Mol Med. 2026 Mar 17;57(5):130. doi: 10.3892/ijmm.2026.5801 (PMC13034893; doi:10.3892/ijmm.2026.5801)

Figure S1. Flow diagram of the literature search and study selection process.

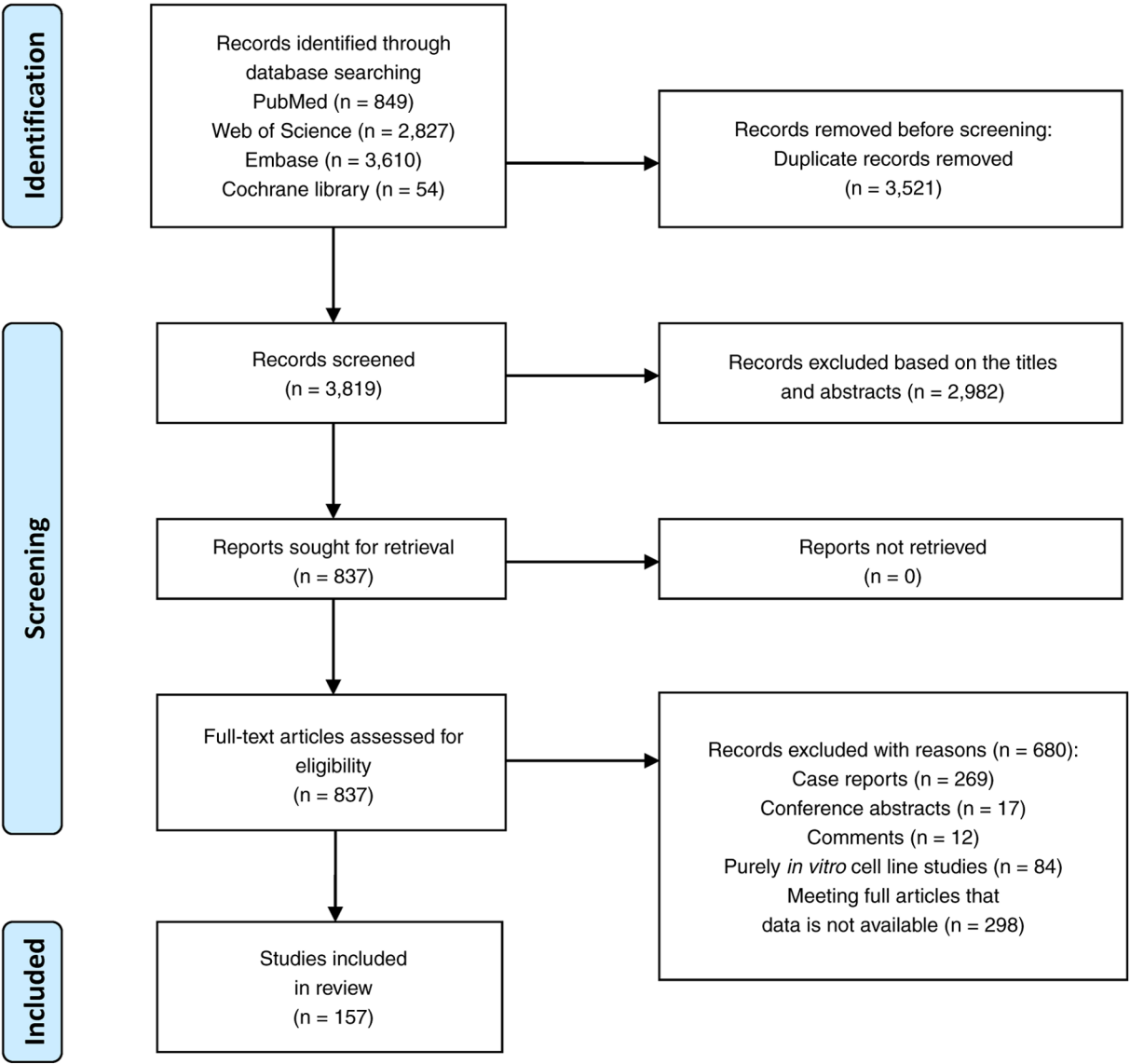

Supplement: Supplementary file 1 [file IJMM-57-5-05801-Supplementary_Data1.pdf]
