# Supplementary material for: The roles and heterogeneity of CD8+ T cells in inflammatory bowel disease: A narrative review of insights from single-cell transcriptomics (Review)
Source: Int J Mol Med. 2026 Mar 17;57(5):130. doi: 10.3892/ijmm.2026.5801 (PMC13034893; doi:10.3892/ijmm.2026.5801)
Supplement: Supplementary file 2 [file IJMM-57-5-05801-Supplementary_Data2.pdf]

Table SI. Search strategy-search terms in the PubMed, Web of Science, Embase and Cochrane Library databases.

| Database | Search terms |
|---|---|
| PubMed | |
| #1 | "Inflammatory Bowel Diseases"[MeSH] OR "Inflammatory Bowel Disease"[Title/Abstract] OR "Bowel Diseases, Inflammatory"[Title/Abstract] |
| #2 | "Crohn Disease"[MeSH] OR "Crohn*"[Title/Abstract] OR "Ileitis*"[Title/Abstract] OR "Enteritis*"[Title/Abstract] OR "Granulomatous*"[Title/Abstract] OR "Ileocolitis"[Title/Abstract] |
| #3 | "Colitis, Ulcerative"[MeSH] OR "Colitis*"[Title/Abstract] OR "Idiopathic Proctocolitis"[Title/Abstract] OR "Inflammatory Bowel Disease, Ulcerative Colitis Type"[Title/Abstract] |
| #4 | #1 OR #2 OR #3 |
| #5 | "CD8-Positive T-Lymphocytes"[MeSH] OR "CD8+T cell"[Title/Abstract] OR "CD8 Positive*"[Title/Abstract] OR "T8 Cell*"[Title/Abstract] OR "T8 Lymphocyte*"[Title/Abstract] OR "CD8-Positive Suppressor T-Cells"[Title/Abstract] OR "Suppressor T Cells, CD8 Positive"[Title/Abstract] OR "T-Cells, CD8-Positive Suppressor"[Title/Abstract] |
| #6 | "Single-Cell Gene Expression Analysis"[MeSH] OR "Single-Cell Gene Expression*"[Title/Abstract] OR "Single-Cell Transcriptome*"[Title/Abstract] OR "Single Cell RNA Seq*"[Title/Abstract] OR "ScRNA seq*"[Title/Abstract] |
| #7 | #5 OR #6 |
| #8 | #4 AND #7 |
| Web of Science | Search terms |
| #1 | TS=((Inflammatory Bowel Diseases) OR (Inflammatory Bowel Disease) OR (Bowel Diseases, Inflammatory)) |
| #2 | TS=((Crohn Disease) OR Crohn* OR Ileitis*OR Enteritis* OR Granulomatous* OR (Ileocolitis)) |

| | |
|---|---|
| #3 | TS=((Ulcerative Colitis) OR Colitis* OR (Idiopathic Proctocolitis) OR (Inflammatory Bowel Disease, Ulcerative Colitis Type)) |
| #4 | #1 OR #2 OR #3 |
| #5 | TS=((CD8-Positive T-Lymphocytes) OR (CD8+T cell) OR CD8 Positive* OR T8 Cell* OR T8 Lymphocyte*) |
| #6 | TS=((Single-Cell Gene Expression Analysis) OR Single Cell* OR Single-Cell Transcriptome* OR Single-Cell RNA Seq* OR ScRNA-seq*) |
| #7 | #5 OR #6 |
| #8 | #4 AND #7 |
| Embase | Search terms |
| #1 | 'inflammatory bowel disease'/exp OR 'inflammatory bowel diseases':ti,ab,kw OR 'inflammatory bowel disease':ti,ab,kw |
| #2 | 'crohn disease'/exp OR 'crohn disease':ti,ab,kw OR 'crohn*':ti,ab,kw OR 'enteritis*':ti,ab,kw OR 'regional enterocolitis':ti,ab,kw |
| #3 | 'ulcerative colitis'/exp OR 'chronic ulcerative colitis':ti,ab,kw OR colitis*:ti,ab,kw OR 'colon, chronic ulceration':ti,ab,kw OR 'ulcerative proctocolitis':ti,ab,kw |
| #4 | #1 OR #2 OR #3 |
| #5 | 'cd8+ t lymphocyte'/exp OR 'cd8 t cell*':ti,ab,kw OR 'cd8+ t lymphocyte*':ti,ab,kw OR 'cd8+ t cell*':ti,ab,kw OR 'cd8-positive t-cell*':ti,ab,kw OR 'cd8-positive t-lymphocyte*':ti,ab,kw OR 't8 cell*':ti,ab,kw OR 't8 lymphocyte*':ti,ab,kw |
| #6 | 'single cell rna seq'/exp OR 'sc rna seq':ti,ab,kw OR 'single cell rna seq*':ti,ab,kw OR 'sc rnaseq':ti,ab,kw OR 'scrna seq':ti,ab,kw OR scrnaseq:ti,ab,kw |
| #7 | #5 OR #6 |
| #8 | #4 AND #7 |

| Cochrane Library | Search terms |
|---|---|
| #1 | (mh "Inflammatory Bowel Diseases") OR (Bowel Diseases, Inflammatory):ti,ab,kw OR (Inflammatory Bowel Disease):ti,ab,kw |
| #2 | (mh "Crohn Disease") OR (Ileocolitis):ti,ab,kw OR (Regional Ileitides):ti,ab,kw OR (Ileitis*):ti,ab,kw OR (Enteritis*):ti,ab,kw OR (Granulomatous*):ti,ab,kw OR (Crohn's Disease):ti,ab,kw OR (Inflammatory Bowel Disease 1):ti,ab,kw OR (Crohns Disease):ti,ab,kw |
| #3 | (mh "Colitis, Ulcerative") OR (Idiopathic Proctocolitis):ti,ab,kw OR (Ulcerative Colitis Type):ti,ab,kw OR (Ulcerative Colitis):ti,ab,kw OR (Colitis Gravis):ti,ab,kw |
| #4 | #1 OR #2 OR #3 |
| #5 | (mh "CD8-Positive T-Lymphocytes ") OR (CD8 Positive*):ti,ab,kw OR (T8 Cell*):ti,ab,kw OR (T8 Lymphocyte*):ti,ab,kw OR (Cells, T8):ti,ab,kw OR (Lymphocytes, T8):ti,ab,kw |
| #6 | (mh "Single-Cell Gene Expression Analysis") OR (Single Cell RNA*):ti,ab,kw OR (ScRNA seq*):ti,ab,kw OR (Single Cell Gene Expression*):ti,ab,kw OR (Single Cell Transcriptome*):ti,ab,kw |
| #7 | #5 OR #6 |
| #8 | #4 AND #7 |
